# Upper pole sleeve fracture of the patella secondary to patellar dislocation: A case report

**DOI:** 10.1097/MD.0000000000016011

**Published:** 2019-06-14

**Authors:** Yingzhi Li, Haichi Yu, Bingzhe Huang, Wei Zhang, Yaxiong Wang, Xiaoning Liu

**Affiliations:** Orthopaedic Medical Center, The Second Hospital of Jilin University, Changchun, China.

**Keywords:** adolescents, patella, sleeve fracture, upper pole

## Abstract

**Rationale:**

Upper pole sleeve fractures of the patella are rare in adolescents; however, they are serious injuries that require early diagnosis and treatment.

**Patient concerns:**

We present a rare case of a 15-year-old girl who suffered a sleeve fracture at the superior pole of the right patella. The patient had a history of dislocation of the patella 2 weeks ago. Physical examination showed tenderness on palpation over the upper pole of the patella and absence of active movement of the knee accompanied by swelling and joint effusion. A plain radiograph showed an avulsed fragment of the superior pole of the patella.

**Diagnoses:**

Magnetic resonance imaging showed a superior pole patellar avulsion fracture and dysfunction of the knee extensor mechanism.

**Interventions:**

Under general anesthesia, the patient underwent open surgery for reduction of the patellar fracture and reconstruction of the knee extension apparatus through an anterior approach.

**Outcomes:**

Six months after the operation, the knee function was fully restored, there was absence of pain and swelling, and the patient was able to return to sports.

**Lessons:**

Upper pole sleeve fracture of the patella is usually serious and it needs to be diagnosed and treated as soon as possible, the sports medicine practitioner must be aware of this type of injury.

## 1 Introduction

Sleeve fractures were first described by Houghton and Ackroyd in 1979, and this type of fracture almost exclusively represents the most common type of patellar fracture in children.^[1]^ This sleeve-like fracture is attributed to premature loss of the immature osteochondral junction in children compared to a fully ossified adult patella.^[2]^ The majority of these injuries are sleeve avulsion fractures of the inferior patellar pole, and they occur due to forced knee flexion or direct violence against the patellar pole.^[3]^ We report an extremely rare case of a 15-year-old girl with a superior pole patellar sleeve fracture that occurred after dislocation of the patella. Previously, only 1 case has been reported in the literature.^[4]^ Patient and her family had provided informed written consent for publication of the case, and the Jilin university second hospital institutional review board approved the study.

## 2 Case report

**Figure 1:**
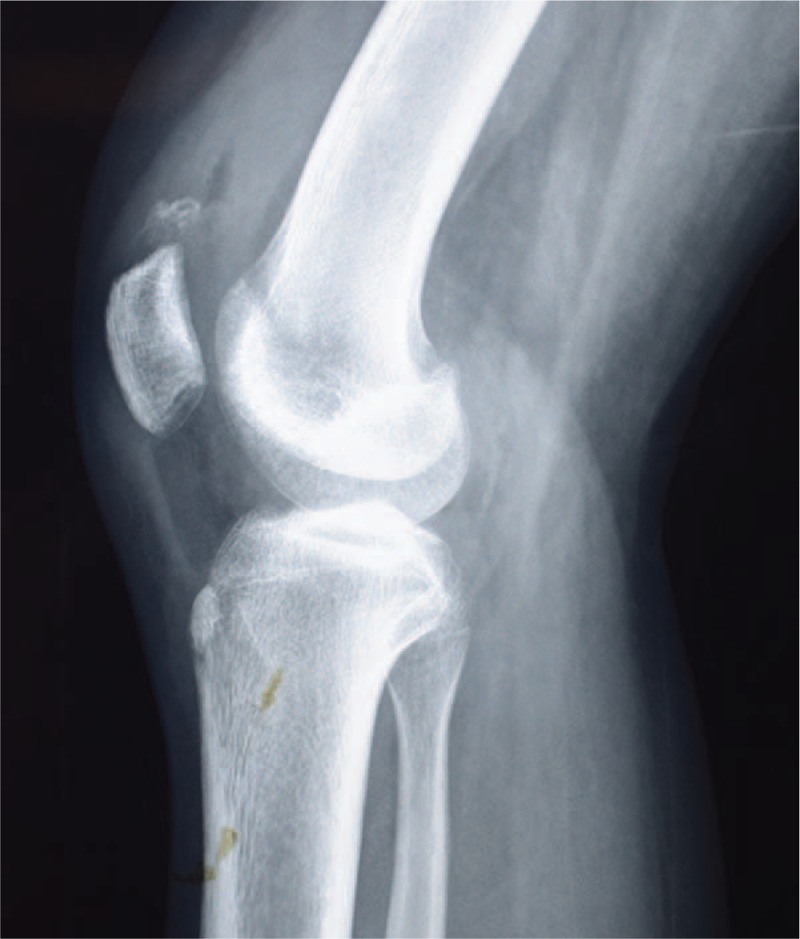
Knee lateral view showed an avulsed fragment of the superior pole of the patella.

**Figure 2:**
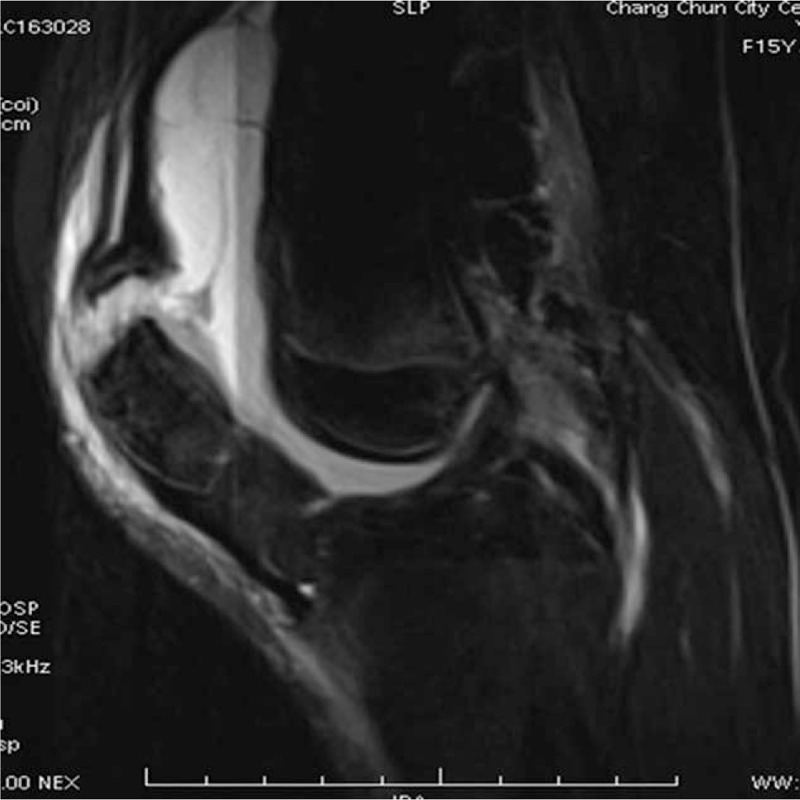
MRI sagittal T2 with fat suppression showed a superior pole fracture of the patella with an intact quadriceps tendon. MRI = magnetic resonance imaging.

A 15-year-old girl visited the orthopedic service because of severe knee pain after a sudden fall while heading downstairs, and she was unable to stretch her leg. The patient had a history of dislocation of the patella 2 weeks ago. Physical examination showed tenderness on palpation over the upper pole of the patella and absence of active movement of the knee accompanied by swelling and joint effusion. A plain radiograph showed an avulsed fragment of the superior pole of the patella (Fig. 1). Magnetic resonance imaging (MRI) confirmed a superior pole patellar avulsion fracture and dysfunction of the knee extensor mechanism (Fig. 2). There were no signs of damage to the articular cartilage, meniscus, and cruciate ligament caused by previous dislocation.

**Figure 3:**
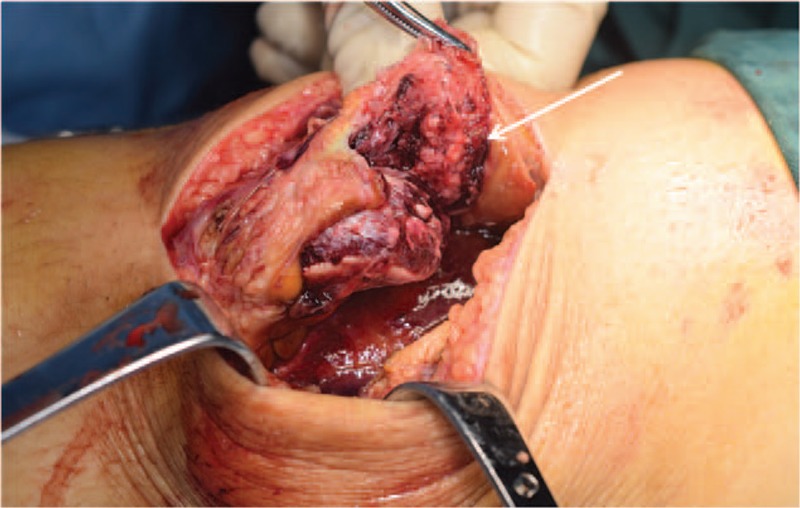
Intraoperative view showed a superior pole fracture of the patella (white arrow) with complete rupture of the patellar retinaculum.

**Figure 4:**
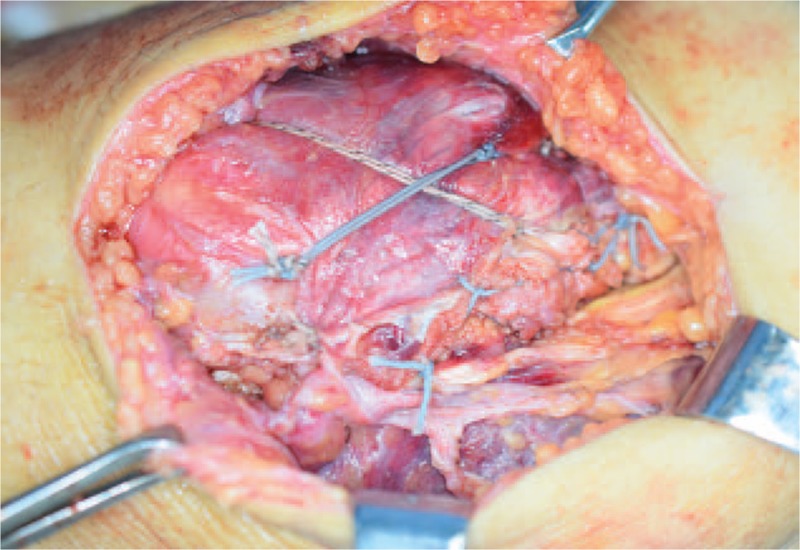
The superior pole fracture of the patella was secured to the main body of the patella by performing augmentation with the figure-of-8 technique.

Under general anesthesia, the patient underwent open surgery for reduction of the patellar fracture and reconstruction of the knee extension apparatus through an anterior approach. All of the above-mentioned imaging findings were further confirmed during surgery. Through a midline incision above the quadriceps tendon, careful dissection was performed to expose the injury site. Intraoperatively, a sleeve-like piece of bone was detected above the patella, and the proximal quadriceps tendon was attached to the bone piece (Fig. 3). Then, complete removal of the blood clot and tissue debris was performed in the joint cavity and around the torn ends of the quadriceps tendon. Patellar retinaculum and quadriceps tendon with the patellar fracture piece was meticulously repaired and secured to the main patellar body by 2 Bio Composite suture anchors (Arthrex, Naples, FL) using a modified Mason–Allen stitch and was augmented by performing the figure-of-8 technique (Fig. 4).

Postoperatively, the patient's knee was maintained in full extension using a knee brace, and weight-bearing was prohibited until 6 weeks after surgery. The passive range of motion exercise of the knee was started on the first day after surgery, and it was gradually progressed to full-scale motion as the tolerance improved. During the first 6 weeks of surgery, the patient's main activities included patellar mobility exercises, knee passive range of motion exercises, and quadriceps activation exercises. After 6 weeks, the patient was referred to the rehabilitation department for restoration of complete knee activity and initiation of muscle strength training. The patient was allowed to gradually resume sports when the normal strength, stability, and knee range of motion were comparable to those on the contralateral side.

Six months after the operation, the knee function was fully restored, there was absence of pain and swelling, and the patient was able to return to sports.

## 3 Discussion

A sleeve fracture of the patella is a unique type of fracture, which accounts for 57% of the fractures of the patella in skeletally immature patients.^[3,5]^ The influence of age on this type of injury may be due to the presence of a more flexible ligament in children; hyaline cartilage and joint capsule cause general joint laxity that protects the patella from trauma. Therefore, it is often found that this type of injury results in an avulsion fracture of the subchondral bone together with that of the cartilage and retinaculum rather than tendon rupture.^[5,6]^ Sleeve fractures of the patella in adolescents usually occur at the lower pole of the patella, and they rarely involve the upper pole of the patella. The present case is unique as it shows a superior pole patellar sleeve fracture secondary to dislocation of the patella. Because it is a rare type of injury, only 1 case has been reported in the literature by Kumar and Knight.^[4]^ In their report, a 14-year-old girl sustained a superior pole patellar fracture 2 days after removal of the knee cast because of a patellar dislocation event that had occurred 1 month ago.

In spite of the extremely low incidence of patellar sleeve fractures, they are serious injuries that require early diagnosis and treatment. Sometimes it is difficult to make a diagnosis on the basis of physical evaluation since the patient often presents with severe pain and swelling in the knee joint. This is especially true if the plain radiograph shows negative evidence of fracture since it may be a largely cartilaginous fracture. The diagnosis of a patellar sleeve fracture depends on a detailed medical history, an analysis of the injury mechanism, and physical and radiological findings. Severe knee pain, swelling, limited knee extension, and an accessible suprapatellar gap might remind us of the possibility of a patellar sleeve fracture. In this case, MRI was necessary to confirm the diagnosis and the associated injury.

Early open surgical repair is the treatment of choice for such injuries. The literature shows that patients diagnosed and treated within 1 week after the injury have a higher functional knee score.^[5]^ Different surgical techniques including the use of fixation systems, such as suture bridge technique, bone tunneling technique, and tension band technique based on the fragment size and location, in addition to the variable augmentation method, have been introduced.^[3–5,7]^

In summary, we reported a rare case of a young girl who suffered a fracture of the superior patellar pole secondary to dislocation of the patella. This type of injury is usually serious and it needs to be diagnosed and treated as soon as possible. The sports medicine practitioner must be aware of this type of injury.

## Author contributions

**Conceptualization:** Yingzhi Li, Haichi Yu, Bingzhe Huang, Xiaoning Liu.

**Data curation:** Yingzhi Li, Haichi Yu, Bingzhe Huang, Xiaoning Liu.

**Investigation:** Haichi Yu, Bingzhe Huang, Xiaoning Liu.

**Methodology:** Yingzhi Li, Haichi Yu.

**Resources:** Yingzhi Li, Haichi Yu.

**Visualization:** Wei Zhang, Yaxiong Wang.

**Writing – original draft:** Bingzhe Huang, Xiaoning Liu.

**Writing – review and editing:** Bingzhe Huang, Xiaoning Liu.
